# TPMS-based membrane lung with locally-modified permeabilities for optimal flow distribution

**DOI:** 10.1038/s41598-022-11175-y

**Published:** 2022-05-03

**Authors:** Felix Hesselmann, Michael Halwes, Patrick Bongartz, Matthias Wessling, Christian Cornelissen, Thomas Schmitz-Rode, Ulrich Steinseifer, Sebastian Victor Jansen, Jutta Arens

**Affiliations:** 1 Department of Cardiovascular Engineering, Institute of Applied Medical Engineering, Helmholtz Institute, RWTH Aachen University, Pauwelsstr. 20, 52074 Aachen, Germany; 2 Chair of Chemical Process Engineering, RWTH Aachen University, Forckenbeckstr. 51, 52074 Aachen, Germany; 3 DWI-Leibniz Institute for Interactive Materials, RWTH Aachen University, Forckenbeckstr. 50, 52074 Aachen, Germany; 4 Department of Pneumology and Internal Intensive Care Medicine, Medical Clinic V, RWTH Aachen University Hospital, Pauwelsstr. 30, 52074 Aachen, Germany; 5 Institute of Applied Medical Engineering, Helmholtz Institute, RWTH Aachen University, Pauwelsstr. 20, 52074 Aachen, Germany; 6 Chair of Engineering Organ Support Technologies, Department of Biomechanical Engineering, Faculty of Engineering, Technology University of Twente, Enschede, The Netherlands

**Keywords:** Cardiovascular diseases, Biotechnology, Engineering, Biomedical engineering

## Abstract

Membrane lungs consist of thousands of hollow fiber membranes packed together as a bundle. The devices often suffer from complications because of non-uniform flow through the membrane bundle, including regions of both excessively high flow and stagnant flow. Here, we present a proof-of-concept design for a membrane lung containing a membrane module based on triply periodic minimal surfaces (TPMS). By warping the original TPMS geometries, the local permeability within any region of the module could be raised or lowered, allowing for the tailoring of the blood flow distribution through the device. By creating an iterative optimization scheme for determining the distribution of streamwise permeability inside a computational porous domain, the desired form of a lattice of TPMS elements was determined via simulation. This desired form was translated into a computer-aided design (CAD) model for a prototype device. The device was then produced via additive manufacturing in order to test the novel design against an industry-standard predicate device. Flow distribution was verifiably homogenized and residence time reduced, promising a more efficient performance and increased resistance to thrombosis. This work shows the promising extent to which TPMS can serve as a new building block for exchange processes in medical devices.

## Introduction

### Current membrane lungs and limitations in design

Hollow fiber membranes have been the industry standard for a variety of technical and industrial membrane separation processes for decades^1,2^. Many modern medical therapies are based on these kinds of membrane separation processes, directing patients’ blood into a membrane module in order to support native organ function. Therapies such as renal replacement therapy^3^, artificial extracorporeal liver support^4^, or extracorporeal lung assist (ECLA)^5^ are popular treatment options for many patients. While distinct, each of these therapies depends on membrane separation processes. Likewise, while the devices used in each of these therapies have certain unique requirements, adequate exchange efficiency is a ubiquitous design requirement and highly depends on homogeneity of flow through the hollow fiber membrane bundle.

In membrane lungs, velocity inhomogeneities are most commonly the result of inlet and outlet geometries that introduce and receive blood flow to and from the fiber bundle non-uniformly. Overall, this non-uniform flow distribution has several negative impacts on device performance. First, it creates areas of high-velocity flow regimes within the bundle exposing the blood to high shear stresses and causing red blood cell damage and platelet activation^6^. Second, while in stagnation areas saturated blood is not removed, shunt flows may occur leading to insufficient exploitation of the gas exchange surface area^7–9^. This reduces the overall exchange efficiency of the device. Finally, non-uniform flow fields result in areas of low or stagnant flow, which can lead to thrombus formation in the fiber bundle^10,11^. Aside from simply blocking the exchange surface of the device, these thrombi can embolize and lead to mechanical failure of the device or even cause adverse events for the patient^12–15^. Indeed, oxygenator thrombus was found to be one of the leading mechanical complications in extracorporeal membrane oxygenation (ECMO), with one review paper revealing a 20% rate of clotting among 1473 cases^16^.

Modern fiber membrane bundles consist of hollow fiber mats either wound around a central core or stacked on top of one another perpendicularly. In membrane lungs, blood flow is directed around the outer lumen of the fibers, with gas flowing through the fiber interiors. However, blood flowing through a bank of fibers presents a unique challenge. Regardless of their form, relying on hollow fibers as the building blocks of their membrane modules leads to one main consequence for all membrane lungs: a uniform resistance to flow. This uniform resistance is a result of the uniform geometry of the hollow fibers as well as the narrow, equally spaced arrangement of the fiber mats. Another way to consider this would be to discuss the fluid permeability of the fiber bundles as porous media as they are typically modelled for simulation purposes^10,17,18^. Dependent only on the geometry of the flow path, permeability, *K*_*perm*_, relates pressure loss, $\frac{\partial p}{\partial {x}_{i}}$, to superficial velocity, *v*_*s*_, for a particular direction in creeping flows via Darcy’s Law^19^:

1 $$\frac{\partial p}{\partial {x}_{i}}= - \frac{h}{{K}_{perm,i}}{v}_{s,i}$$

where η is the dynamic viscosity. The equation illustrates that the local superficial velocity is directly proportional to the driving pressure gradient via the permeability. This means that a uniform flow resistance, i.e. a constant permeability, cannot compensate for flow inhomogeneities between, for example, two neighboring streamlines. The implication here is that local modification of the permeability would enable influencing flow through microscopic modifications towards a homogeneous macroscopic flow distribution. Ultimately, this would result in more efficient devices that are also less prone to stagnation and thrombosis. Therefore, an alternative to hollow fibers is required to improve the performance and safety of membrane lungs.

### New design opportunities through TPMS-based membranes

Contemporary additive manufacturing technologies have enabled the production of triply periodic minimal surfaces (TPMS) for a wide variety of technical and industrial applications^20–22^. TPMS are surfaces that divide space into two congruent, interwoven compartments. TPMS are periodic and can be infinitely extended in all three spatial directions. As membrane spacers, TPMS geometries improved heat and mass transfer and reduced fouling in ultrafiltration, distillation, and reverse osmosis applications^23,24^. As the macroscopic building blocks of membranes, TPMS geometries increased separation efficiency in oil-in-water demulsification^25^. Initial investigations into the heat and mass transfer properties of TPMS-based microfluidic modules showed significant improvement over traditional membrane geometries^26–28^. Also, for blood-gas exchangers, a higher gas transfer rate compared to a state-of-the-art hollow fiber membrane design was shown experimentally^29^. Aside from their high inter-connectivity and stability, TPMS also can be used to create lattices with porosity gradients^30–32^. While this is often applied in the context of preparing cell scaffolds for tissue engineering, similar techniques could be applied to manipulate the flow distribution of a passing fluid. Integration of TPMS into membrane modules consisting of a network of individually modifiable periodic elements could not only increase the module's efficiency but also help to ameliorate hallmark problems in extracorporeal transmembrane therapies.

Here, we present a novel method of creating TPMS-based membrane modules for improving flow distribution in membrane lungs using a permeability-based approach for manipulating the form of TPMS. A simulation-based optimization scheme for determining the distribution of permeability throughout a membrane module was developed. The results of said simulation were then translated to the design of a prototype membrane module. Finally, prototype devices were manufactured and compared to a contemporary membrane lung device using in vitro experiments to validate both simulation results and the TPMS-based membrane geometries.

### Fundamentals of this study

In order to rigorously evaluate the effect of the locally modified novel membrane geometries on the flow distribution in a membrane lung, a market standard device was chosen as a comparative model. The Novalung interventional Lung Assist (iLA) device (Xenios AG, Heilbronn, Germany) is often indicated in patients with respiratory acidosis as a result of acute respiratory distress syndrome (ARDS)^33^. It is approved for flows between 0.5 and 4.5 L/min, and has been shown to maintain a pressure drop below 20 mmHg over the entire flow range^34^. This low resistance allows the iLA to be used in pumpless applications driven by the patient’s arterio-venous pressure gradient and thus, at relatively low flow rates^35^. Low flow applications clearly enhance the risk of thrombus formation, highlighting the importance of a homogeneous flow distribution within the device.

**Figure 1:**
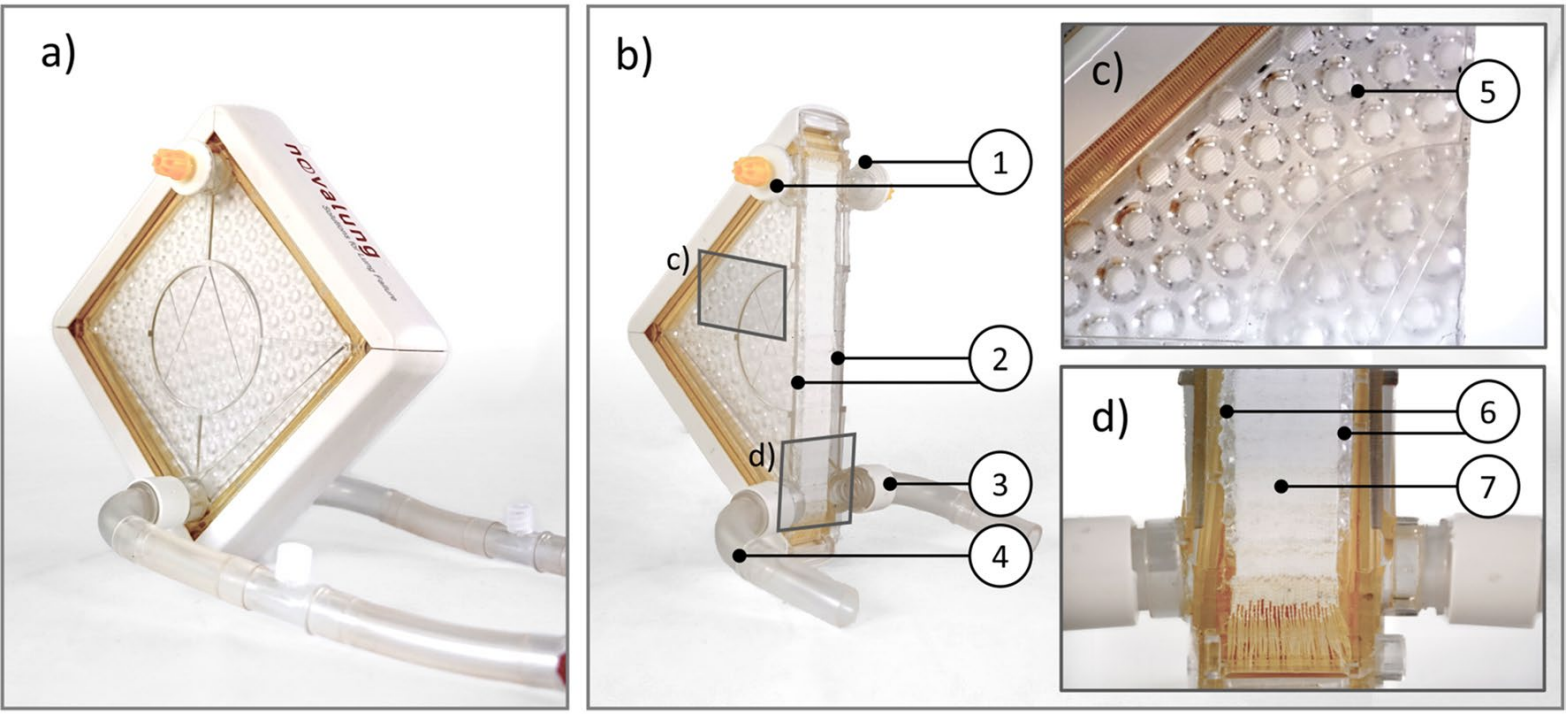
A iLA Membrane Ventilator used as predicate device in this study (**a**) Overview of the predicate device. Note the opposing inlet and outlet (**b**) cut-open predicate device (1) de-airing ports in the uppermost corner (2) antechambers (3) outlet (4) inlet [rectangles indicate postion of the magnified images in (**c**) and (**d**)] (**c**) magnified side-view (5) distributor plate with equally spaced holes (**d**) magnified view of inlet–outlet section (6) distributor plates (7) hollow fiber bundle.

The device consists of two identical flow antechambers placed on either side of a stacked fiber mat (Fig. 1a). The fiber bundle itself constitutes a 1.3 m^2^ exchange area made up of polymethylpentene (PMP) hollow fibers stacked perpendicularly to one another, forming a criss-cross pattern. The inner and outer diameters of the PMP hollow fibers (OXYPLUS™, 3 M/Membrana, Wuppertal, Germany) are 200 µm and 380 µm, respectively. A more detailed description of the fiber arrangement in such a hollow fiber membrane bundle was published elsewhere^36^. As a result, the fiber bundle displays two distinct permeabilities: streamwise, normal to each fiber mat; and transverse, along the length of either set of fibers. The bundle has a footprint of approximately 100 × 100 mm^2^ and is 20 mm thick. A 2 mm thick polycarbonate diffuser plate, containing a hexagonal pattern of 4 mm holes, is placed on each side of the fiber bundle (Fig. 1c,d). The inlet and outlet are directly opposite to each other in the lower corner of the device (Fig. 1b). In the upper corner, de-airing port allows for easy priming of the device during initiation of therapy. While this improves the usability of the device, it also favors an uneven flow distribution during operation. While in the lower corner shortcutting of the flow between inlet and outlet is very likely, in the upper corner the risk of stagnated flow and thrombosis is elevated, especially under low flow conditions (< 1.5 L/min)^37,38^. Indeed, initial experiences with the iLA device indicated that areas of the flow chambers with low or stagnant flow were precisely the areas in which the majority of thrombi were found^34^.

The aim of this study is to develop a TPMS-based prototype based on the iLA device, providing ideal homogenous flow conditions. Under inhomogeneous flow conditions, streamwise flow velocities deviate substantially from the average, referred to here as *v*_*ideal*_. By contrast, homogenous flow conditions are achieved as this deviation from *v*_*ideal*_ becomes zero.

**Figure 2:**
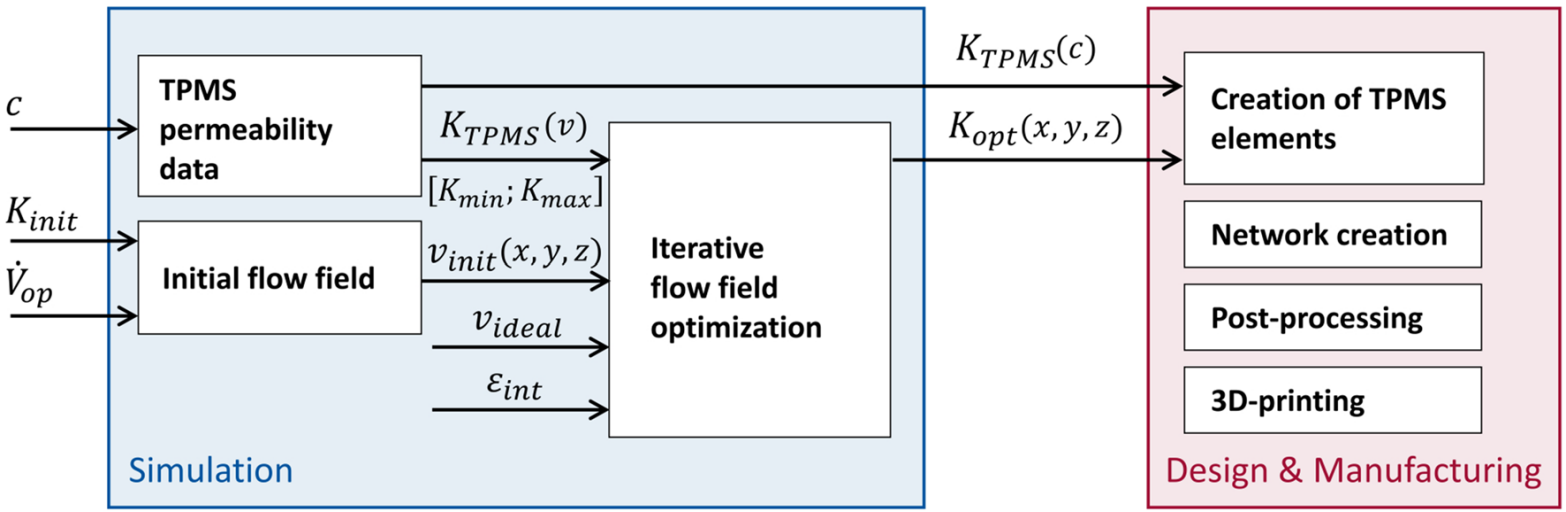
Schematic workflow used in this study for remodeling a predicate device towards optimized flow distribution based on TPMS membrane elements. In subsequent design and manufacturing, the geometric relation of the TPMS elements and their permeability K_TPMS_(c) is used for the translation of the optimized permeability field K_opt_ into real-world TPMS models. The single surfaces are then assembled into a network and prepared for 3D-printing.

The presented approach includes several steps of iterative flow simulations and post-processing to fabricate such prototypes. Before going into detail in the following sections, an overview of how the prototype was designed and constructed is provided here (Fig. 2). First, baseline simulations of the initial flow field of the predicate device were conducted in order to provide a comparative basis for subsequent prototype simulations. The initial flow field *v*_*init*_ was simulated using an operational flow rate (${\dot{V}}_{op}$) and uniform permeability (*K*_*init*_) of the fiber bundle domain. Then, simulations of individual TPMS elements were conducted to determine the achievable range of membrane module permeabilities. Practically, this was done by warping standard TPMS geometries by a multiplicative factor ‘*c*’ and then simulating flow across these deformed TPMS. The resulting pressure losses for each geometry were used to calculate the range of achievable permeabilities [*K*_*min*_; *K*_*max*_], which were then used to inform the bounds of an optimization process. In that process, flow velocities through a prototype module were simulated, compared to an ideal scenario, and used to update the 3D pointwise permeability in the device for the next iteration. In praxis, this means the optimization scheme manipulated the pointwise permeability within the limits of [*K*_*min*_; *K*_*max*_] according to the difference between the simulated flow velocity at that point and the ideal flow velocity *v*_*ideal*_*.* This updated, non-uniform permeability field *K*_*opt*_*(x,y,z)* was then used in the next iteration, instead of *K*_*init*_. This iterative process was continued for a single flow rate until simulation results showed sufficient improvement compared to the predicate device. Afterwards, the prototype device was simulated at each of the flow rates chosen for the predicate device to allow for appropriate comparison and analysis of the results.

At this point, the 3D dataset representing the pointwise permeability *K*_*opt*_*(x,y,z)* was exported and translated to CAD data. This spatially defined permeability information needed to be translated into TPMS geometry data which could then be used to create prototype membrane modules that would exhibit the prescribed local permeabilities and could be printed using rapid prototyping methods. The relation between the TPMS elements’ geometry and their permeability, *K*_*TPMS*_*(c),* was used to do this. A corpus of STL files was created representing the TPMS elements which would be necessary to create the prescribed membrane module. These STL files were then combined, post-processed, and prepared for 3D printing. The membrane modules were 3D printed and assembled along with peripheral components into the prototype device, which was subsequently used for benchtop tests.

## Results

**Figure 3:**
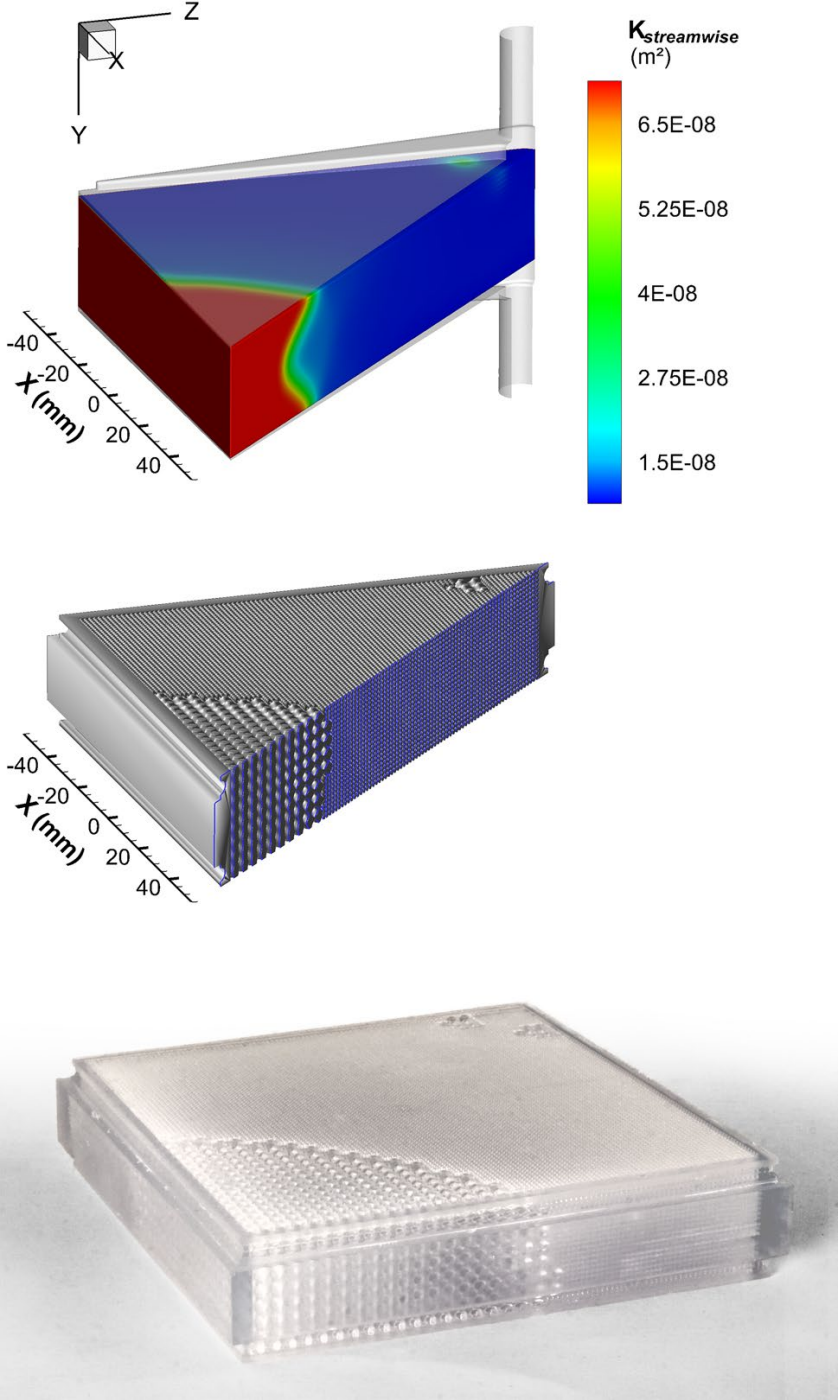
Concretization steps of the TPMS-based membrane module with locally modified permeabilities. (**a**) Simulated permeability field, (**b**) STL prototype after translation of the permeability into Schwarz-P (SWP) TPMS elements including the additional frame, (**c**) 3D printed membrane module. Flow direction in all images is in positive y-direction.

**Figure 4:**
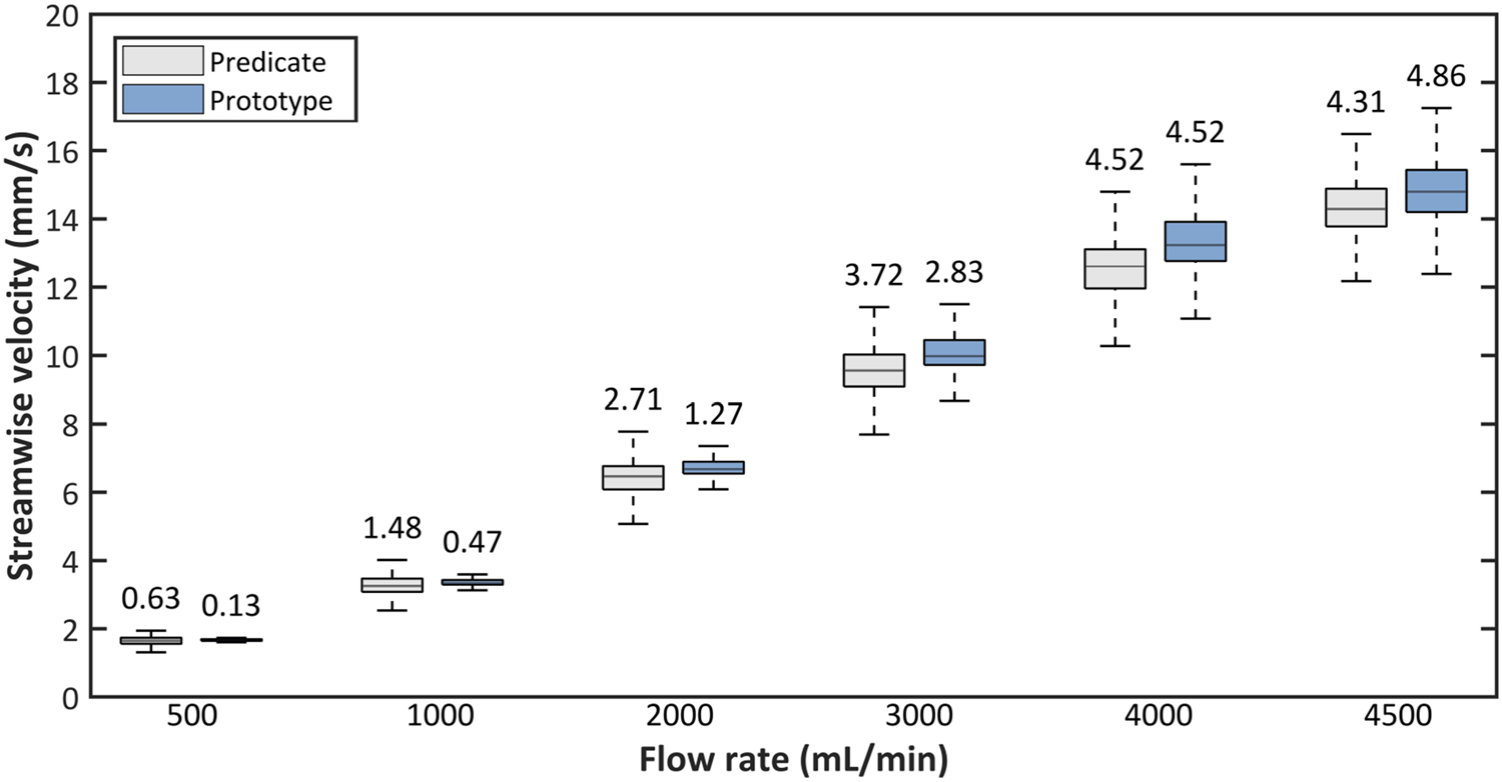
Box and whisker plots of streamwise flow velocities in the membrane modules of the predicate and prototype devices. Midlines show the median value, boxes cover the 25th to 75th percentile, and whiskers show minimum and maximum. The values above the plots of the predicate and final prototype indicate the velocity range between the top and bottom whisker.

Figure 3a shows the results of the optimization scheme in form of the streamwise permeability field after 176 iterations. Multiple factors were considered in order to reach this decision. After 176 iterations, the streamwise component of the velocity distribution showed significant improvement over the predicate device in terms of homogeneity of flow velocities (visible in Fig. 4 as the boxplot at 1 L/min). Additionally, the volume fraction of the module with flow velocities lower than 3 mm/s had leveled off, implying a lack of possible additional improvement. Aside from two small areas of high permeabilities directly neighboring where flow entered the membrane module, the overall module had a gradient from low to high permeabilities as one gets farther away from the inlet (Fig. 3a). The permeability data translated into a membrane module is depicted in Fig. 3b. The final prototype is shown in Fig. 3c.

Figure 4 compares the streamwise velocity components of flow through the membrane modules at each simulated flow rate. The middle lines of each box depict the median velocity, the boxes extend to the 25th and 75th percentiles, and the outermost lines represent the range of the most extreme data points. At 1 L/min flow rate, the design point for the iterative optimization, the range for the prototype was successfully narrowed to 0.47 mm/s compared to 1.48 mm/s. Over the full flow range, the range between maximum and minimum velocities in the predicate module varied between 0.63 mm/s for the lowest flow rate to 4.52 mm/s for the second-to-highest flow rate. In the simulated prototype module, this range was 0.13 mm/s for the lowest flow rate and 4.86 mm/s at the highest flow rate. Like in the predicate module, the range between maximum and minimum velocities in the prototype consistently increased with increasing flow rate except for the highest flow rate. At the highest flow rate, a decrease is observed. The range of flow velocities was narrower in the final prototype device than in the predicate at all flow rates below 4000 mL/min. At 4000 mL/min the two devices performed equally, and at 4500 mL/min the predicate device created a slightly narrower range of flow velocities. Also, the median velocities in the prototype were consistently higher than that of the predicate, as the volume porosity of the prototype (0.5) was slightly higher than the porosity of the predicate (0.493).

**Figure 5:**
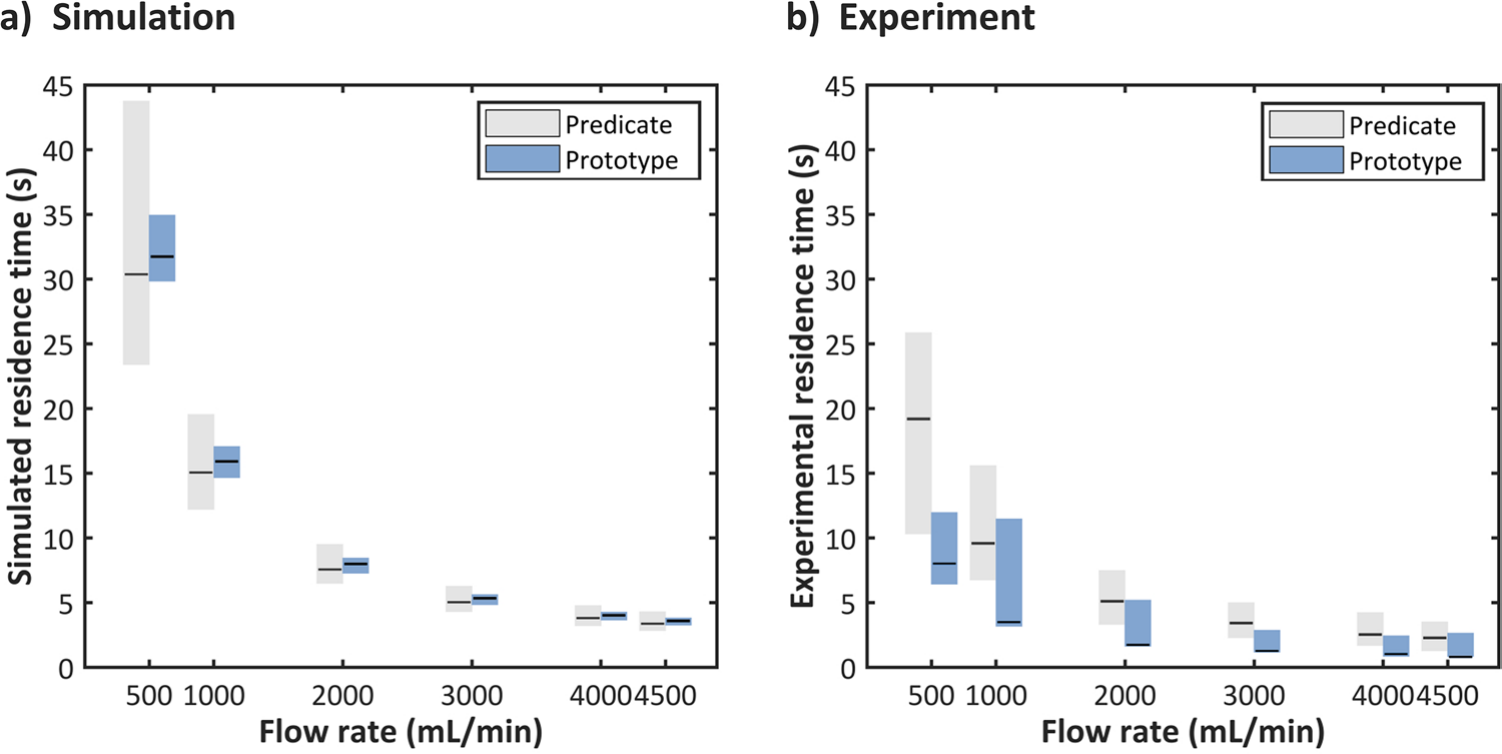
Residence times depicted as mean value with minimal and maximum span: (**a**) calculated by simulation and (**b**) measured in experiment.

While these velocities can be directly quantified in simulations, real-world conditions precluded observing the flow within the fiber bundle. Instead, the residence time of fluid in the device was measured as an analog for flow velocity. Figure 5 shows (a) the simulated and (b) experimental minimum, mean, and maximum residence times of the predicate and prototype devices at each flow rate. In both the predicate and prototype simulations, the range between minimum and maximum residence times consistently decreased as the flow rate increased. However, these ranges were consistently smaller in the prototype device. While the mean value of the prototype is slightly higher at every flow rate, the range between minimum and maximum value is narrower over the full tested range.

The experimental mean residence times for the prototype on the other hand are all lower than for the predicate. The range of residence times of the prototype are also narrower than that of the predicate. The duration between the minimum and maximum residence times for the predicate device ranged from 15.59 s at the lowest flow rate to 2.3 s at the highest flow rate, and the time spans consistently decreased as the flow rate increased. For the prototype device, durations spanned from 8.35 to 1.64 s, but here a consistent decrease in relation to increasing flow rate was not observed. The longest duration occurred at the 1 L/min flow rate, and the shortest duration was observed at 4 L/min. While the minimum and mean residence times for the prototype consistently decreased with increasing flow rate, the maximum residence time was slightly more erratic.

## Discussion

This study suggests a practical approach based on the unique property of a TPMS-based membrane network with elementwise modification of the local permeability to achieve a global homogenous flow distribution in a membrane lung. Overall, the simulation-based performance of the final prototype device speaks to the validity of the optimization scheme. Figure 4 shows that performing the optimization scheme at one design point (1 L/min) led to a module which performed better than the predicate at multiple other design points. A module modified for a certain flow rate did not reduce its effectiveness for another. However, it can also be seen in the simulation results that the relative improvement compared to the predicate declines as the flow rate increases, to the point where the predicate device slightly exceeds the final prototype at the highest flow rate. Therefore, it could be suggested that the optimization scheme applied here is not independent of flow rate.

The TPMS-based membrane module with locally modified permeabilities achieved a narrower range of minimum and maximum flow velocities in the membrane module. This means that, by leveling these velocities, the flow in stagnation zones was increased and the flow in high flux regimes was reduced. Improved flow homogeneity is widely discussed in literature and promises to overcome two hallmark problems in ECMO therapy: inefficient gas exchange and membrane thrombosis. Hence, the focus of this study was the exploration of a systematic strategy towards a design fulfilling these requirements. Still, the validity of these promises with regard to thrombotic risk and gas exchange is yet to be proven by experiment. For an investigation of the efficacy of flow homogenization on gas transfer, a module based on this design must be developed that is capable of gas exchange. Furthermore, investigating the efficacy of flow homogenization on thrombotic risk requires a prototype module developed according to the industrial standards used for the predicate. Differences in the choice of material, sterilization and manufacturing processes may affect thrombotic risk. The current gold standard for the assessment of thrombotic risk in membrane lungs are animal trials due to the lack of reliable in-vitro test methods. Animal testing, however, are per se very complex and must be performed multiple times to allow a reliable statement due to the potentially high biological variance and the high bandwidth of potential influences.

In simulation results, the prototype device consistently showed reduced pressure loss in comparison to the predicate (predicate: 0.9 mmHg at 500 mL/min up to 17.6 mmHg at 4500 mL/min; prototype: 0.76 mmHg at 500 mL/min up to 13.62 mmHg at 4500 mL/min). This can be attributed to the removal of the distributor plates and the general increase in permeability in the final prototype. The median streamwise permeability of the final prototype device was 2.41e−9 m^2^, compared to 10.88e−10 m^2^ for the predicate device. However, these results were not reflected in the benchtop experiments. There, the predicate device still showed a slightly lower pressure loss at all flow rates (predicate: 1 mmHg at 500 mL/min up to 21 mmHg at 4500 mL/min; prototype: 3 mmHg at 500 mL/min up to 31 mmHg at 4500 mL/min). This is possibly the result of the quality of the 3D print. Due to the limit of the resolution of the machine used, each individual element was slightly larger than prescribed in the CAD model. This global enlargement would result in a higher resistance to flow throughout the entire bundle, thereby raising the pressure loss.

In simulation results, the final prototype also led to a narrower band of residence times for all design points compared to the predicate device. Experimental results confirmed the improvement of residence times within the prototype device at all flow rates. However, while simulation results do qualitatively reflect the trends observed in reality, they overestimate the absolute values of all residence times and underestimate the range of residence times. This could be due to decisions regarding the threshold values for determining real-world residence times (e.g. choosing 95% instead of 99% for the threshold of maximum residence time), but it is most likely due to simplification of the membrane domain in the CFD simulations. Additionally, while the mean residence times of the predicate device lie approximately halfway between the minimum and maximum values, the mean residence times of the prototype is almost identical to the minimum. This could be the result of a number of different factors. First, it could simply be that an overly large portion of the flow was shunted through the prototype device, meaning that the largest single bolus of ink that passes by the second color sensor is also the first. On the other hand, this could also be caused by the aforementioned 3D printing inaccuracies.

The improvements observed were achieved without any additional flow guiding structures. By changing only what would be the gas exchange membrane in the device, not only could the flow distribution be improved compared to modern devices, but the overall hemocompatibility of the device could be markedly improved by reducing the foreign surfaces presented to the blood cells. The two distributor plates represent a total surface area of approx. 17,000 mm^2^ in a device whose non-membrane surface area is approx. 20,000 mm^2^ (not considering the tubes leading to the device or the surface of the potting material). Also, the areas directly behind the diffuser plates are prime areas for flow recirculation and thrombus formation. Removal of the plates represents an 85% decrease in foreign non-membrane surface area and, consequently, was a design goal for the prototype device. Despite improvements in surface coatings used in extracorporeal circuits^39^, the reduction of foreign surface exposure will remain a clear design goal for extracorporeal devices. Novel approaches such as those presented here represent a promising method of using one function-critical component (the exchange membrane) to fulfill additional ancillary functions, reducing the number of components.

When integrating TPMS geometries as membrane elements, consideration should be given to the size required for the individual elements in order to achieve appropriate mass exchange. While the prototype presented here only consisted of what would be the blood channel, it is important to consider the overall surface area of the device for future membranes. With the given size of SWP elements and the outer dimensions matching that of the iLA, the prototype possessed a would-be exchange area of approximately 0.52 m^2^, compared to 1.3 m^2^ for the iLA. One strategy to increase the volume-specific surface area of the prototype would be to use smaller TPMS elements, but here one quickly comes across technical limitations in terms of manufacturability. For example, in order to achieve the same volume-specific surface area of a 380 µm hollow fiber, the bounding box of an individual TPMS element would need to be less than 0.6 mm in every dimension. While certain advanced additive manufacturing techniques can certainly create geometries this small, the ability to accurately produce such fine structures at such large build volumes necessary for an entire prototype is firmly outside the capabilities of modern technologies^20^. Relating to this, additive manufacturing techniques will have to evolve to enable the fabrication of the suggested design from this study as functional gas exchanger including a gas compartment using hemocompatible materials.

It is important to note that the work here addresses only one TPMS, the Schwarz-P surface. During preliminary investigations, it was determined that the permeabilities of the Schwarz-D and Schoen-G could not be varied widely enough to justify further testing with the method presented here. To clarify, the ratio of streamwise permeability between the most occluded and most permeable variants of Schwarz-P surfaces within a given element size was roughly 100, whereas for Schwarz-D and Schoen-G surfaces, this ratio was approximately 5 and 2, respectively. However, there exist strategies for hybridizing these different TPMS in case this might be beneficial for gas transfer performance^32^.

The method here is described as a “permeability-based approach” because the permeability of elements was changed without affecting the overall porosity of any element. This presents some novel and possibly advantageous differences when held against porosity-graded scaffolds, where the constant level offset of the implicit equation is manipulated. In those methods, the permeability of the resulting methods can also be manipulated, but the streamwise and transverse permeabilities of the element remain equal. However, if the elements directly between the inlet and outlet of the devices were to have equal transverse and streamwise permeabilities, lateral motion through the modules could be significantly hampered, which might impede the device’s ability to wash out the outer portions of the module. In fact, keeping the overall volume of the element constant means that any decrease in streamwise permeability must lead to an increase in transverse permeability.

Here, we have proven as hypothesized that local modification of permeability could direct flow on a microscale level towards a fully homogeneous global flow distribution. This was achieved by remodeling a contemporary membrane lung with disadvantageous (from a fluid mechanics point of view) inlet and outlet positions. A goal for future studies should be to investigate to what extent a flow path can be influenced or even redirected by microscopic local variations. This would allow for the design of flow paths through a TPMS-based membrane module without any restriction due to inlet and outlet position. Moreover, methods for defining novel TPMS are the subject of ongoing research. Implicit surface modeling methods enabled the creation of minimal surfaces which were not periodic in the Cartesian coordinate system but rather in a tetrahedral coordinate system and have proven more reliable in replicating complex structures without the need for complex mapping techniques^40^. Such surfaces could provide benefits when designing modules that do not conform to highly-regular geometries like those used in modern oxygenators, possibly even taking the contours of natural organs into account^41^. Independence from inlet and outlet positions and the possibility to efficiently fill organ-shaped voids with TPMS structures would represent tremendous steps towards an implantable artificial lung^41–43^.

Despite successfully establishing and applying a practical approach towards improving flow distribution in a membrane lung, this study has several limitations. Throughout the workflow from computational model to real-world module there are multiple “translation” processes that might cumulatively account for the deviation of the in vitro results from simulation results. First of all, it should not be assumed that the simulation results themselves were completely accurate. The nature of porous domain modeling and the relying on user-defined monitor points over simulation residuals introduces a certain amount of doubt in the accuracy of the results. Secondly, translating the relatively continuous 3D permeability data into a collection of discrete TPMS module elements results in a certain amount of downsampling and interpolation of the permeability. Even though the permeabilities within a 3 mm × 3 mm × 3 mm region might differ from one another, the mean value was calculated and assigned to a single element which then defined the permeability throughout the entire block. Thirdly, the connections between individual TPMS elements represent regions where the permeability was not validated by simulation. Even though the difference in permeability between neighboring elements was often minuscule, the transitions between elements of different sizes might have indeed introduced unanticipated flow patterns through the module that had an effect on the overall performance of the device. Lastly, and perhaps most critically, the resulting form of the individual elements is obviously always dependent on the resolution of the 3D printing process.

## Conclusion

Overall, the simulation-based optimization scheme presented here was effective in creating a design that performed well in benchtop experiments. TPMS elements of varying shapes and sizes could be easily combined to construct a prototype module that contained varying streamwise and transverse permeabilities. A straightforward workflow was developed through which variable distributions of permeabilities in a simulation domain could be established and automatically updated towards a device design. The results of this workflow were accurately translated into prototype CAD models by way of self-developed scripts. While further advancements in manufacturing technologies are necessary in order to effectively translate simulation-based designs into accurate real-world devices, the prototype here performed well when compared against an industry-standard predicate. Based on the presented metrics, our prototype showed an improved flow distribution throughout the membrane module. This work supports the notion that TPMS are a promising tool for advancements in membrane technology, and future work will show the extent to which their integration can lead to safer and more efficient mass transfer in medical devices.

## Methods

### Simulation

All simulations were executed using Ansys CFX 19.0 (Ansys, Inc, Canonsburg, PA, USA). Simulation domains were isolated from CAD models using Ansys SpaceClaim, and all computational meshes were created using Ansys Meshing.

#### TPMS permeability data

Deforming individual TPMS elements facilitated the control of local permeability in the membrane module. In this study, the Schwarz-P (SWP) shape was used. The surface *F*_*SWP*_ of a single SWP element is described by the following implicit equation:

2 $${F}_{SWP}\left(x,y,z\right)= \mathrm{cos}\left(x\right)+\mathrm{cos}\left(y\right)+\mathrm{cos}\left(z\right)=0$$

Under normal conditions, the coefficients of each of the cosine terms of the SWP implicit function are equal to 1. Increasing the coefficient preceding a cosine term for a particular axis decreases the smallest cross-sectional area for fluid flowing around the element along that same axis. However, the overall volume within the unit cell enclosed by the surface remains the same, therefore the volume porosity of the element remains the same.

**Figure 6:**
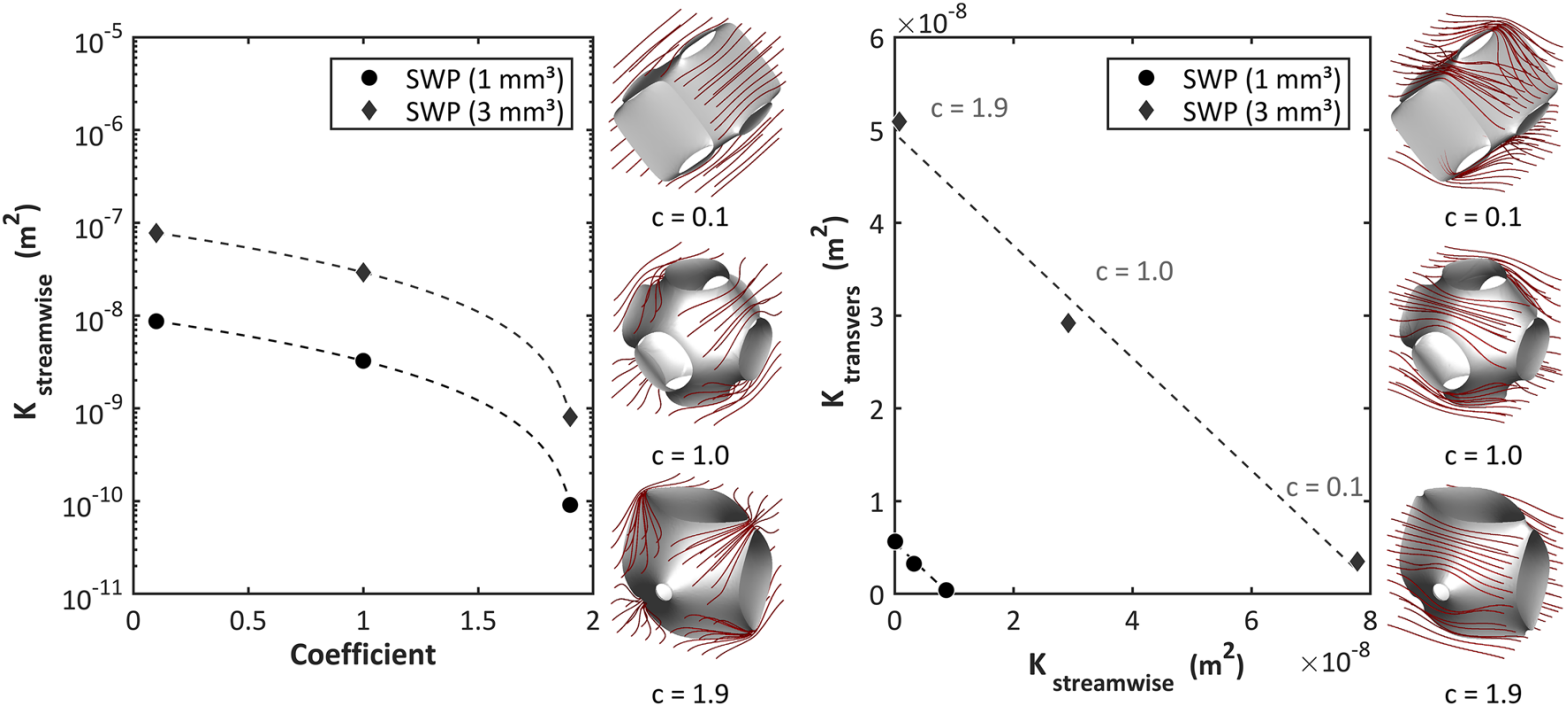
Simulated SWP permeability data: (**a**) Streamwise permeability depending on the cosine. Dashed lines show quadratic correlation curves between the value of the coefficient ‘c’ of the implicit function term and the consequent permeability. Images next to graph give an impression of the undeformed (c = 1.0) and deformed SWP elements. Streamlines indicate streamwise flow direction. (**b**) Relation between streamwise and transverse permeability. Dashed lines show linear correlation curves. Images next to graph give an impression of the undeformed (c = 1.0) and deformed SWP elements. Streamlines indicate transverse flow direction.

The SWP element was simulated to determine the Darcy permeability for several geometries. The simulation was set up to replicate the experimental procedure described in Schlanstein et al.^36^. Low flows were spread over theoretical modules created by translationally periodic boundary conditions, and pressure loss was calculated across the thickness of one layer of the module. In subsequent post-processing, the streamwise and transverse permeabilities were calculated for the standard as well as the “most permeable” (c = 0.1) and “most occluded” (c = 1.9) SWP geometries, illustrated in Fig. 6. The degree of occlusion (or inversely, the available cross section) to the flow determined the permeability of the element. For example, for the most permeable streamwise SWP geometry, a higher cross-sectional area for flow is available associated with lower pressure losses than for the most occluded, streamwise configuration.

To obtain a wide range of permissible permeability, a combination of 1 mm and 3 mm SWP elements was conceived for the bundle. Figure 6a shows the range of streamwise permeabilities that were achievable with both element sizes for the different states of deformation. The relationship between the streamwise and transverse permeabilities of 1 mm and 3 mm elements can be seen in Fig. 6b. The linear regression was used in the optimization scheme. As the design intent was to facilitate as many 1 mm elements in the module as possible, the threshold value for the decision to use 3 mm elements instead of 1 mm elements was set to the upper limit of the 1 mm elements’ streamwise permeability range (8.68e−9 m^2^).

#### Initial flow field

A geometry corresponding to that of the Novalung Interventional Lung Assist (iLA) was simulated to establish a comparative baseline. The simulation geometry was created in Creo Parametric 4.0 (PTC, Boston, MA, USA). All dimensions for the geometry were measured on a disassembled iLA device (Fig. 1b). The fiber bundle was modeled as a porous domain with a constant porosity of 0.493 and an anisotropic permeability. Streamwise permeability was set to 10.88e−10 m^2^, and transverse permeability was set to 7.71e−10 m^2^ in accordance with previously reported data for permeabilities of stacked fiber mats^36^.

The computational domain of the TPMS-based prototype device was identical to that of the predicate device except for the removal of the distributor plates and the widening of the membrane module to occupy the resulting empty space. Removing the distributor plates also meant that a symmetrical boundary condition could be assumed along the diagonal plane from bottom to top of the prototype device, reducing computational effort. Blood was modelled as a Newtonian fluid with a density of 1059 kg/m^3^ and a dynamic viscosity of 3.6 mPas. Flows of 0.5, 1, 2, 3, 4, and 4.5 L/min were tested, corresponding to the range of flow rates for which the iLA is clinically approved.

#### Iterative flow field optimization

With the range of achievable permeabilities in hand, the goal of the iterative optimization scheme was then to determine how those permeabilities should be distributed in the porous domain in order to satisfactorily distribute blood flow throughout the module, creating an optimized flow field. An optimized flow field in this context meant a flow field with homogeneous flow velocities. Practically speaking, between two iterations, the optimization algorithm would lower the permeability at every point in the fiber bundle where the velocity was too high and raise the permeability at every point where the velocity was too low. This iterative process was stopped once the difference between the variable velocity *v*_*y*_ and the ideal velocity *v*_*ideal*_ was acceptably small or the change in difference between two consecutive iterations stagnated. This meant that the simulation workflow needed to (a) prescribe the value of streamwise and transverse permeability over the porous domain according to data provided from the previous iteration, (b) calculate the desirable value of the streamwise permeability based on the deviation of the real flow to the desired flow, and (c) repeat, using the updated permeability values as the provided data in step (a).

After a simulation solution for the given iteration was found, a correction function was defined that determined the new pointwise permeability value to use for the following iteration. The correction function took the form of a hyperbolic tangent function:

3 $${K}_{new}= {K}_{old}\left(1+\mathrm{tanh}\left(p\bullet \left({v}_{ideal}- {v}_{y}\right)\right)\right)$$

where *K*_*new*_ is the streamwise permeability for the next iteration, *K*_*old*_ that of the current iteration, *v*_*ideal*_ is the ideal flow velocity in streamwise direction, *v*_*y*_ is the streamwise flow velocity in the current iteration, and *p* is a constant used to adjust the rate of change between iterations. A 1 L/min flow rate was used for all iterations of the optimization process. The membrane module of the prototype device, like that of the iLA, had a footprint of 100 mm × 100 mm. Accounting for the volume porosity of the SWP elements (0.5), this gave an area average velocity *v*_*ideal*_ of 3.33 mm/s. Initially set at 150, this *p* constant was slowly decreased to 1 as the iterative process proceeded in order to balance rapid progress towards the ideal result with higher precision once the given solution approached the ideal.

In general, it is helpful to establish meaningful initial conditions for the distribution of permeability a priori in order to enhance convergence. Here, a 3D permeability matrix was used to define the streamwise permeability K_init_ at each point in the computational mesh based on the following equation:

4 $${K}_{init}= 5.46\times {10}^{-7}m \cdot {r}_{corner}+ 7.10 \times {10}^{-11} {\text{m}}^{2}$$

where r_corner_ (m) is the distance from the lower corner. The dependent transverse permeability was then defined from the streamwise permeability (see Fig. 6b).

### Design and manufacturing

#### Creation of SWP elements

**Figure 7:**
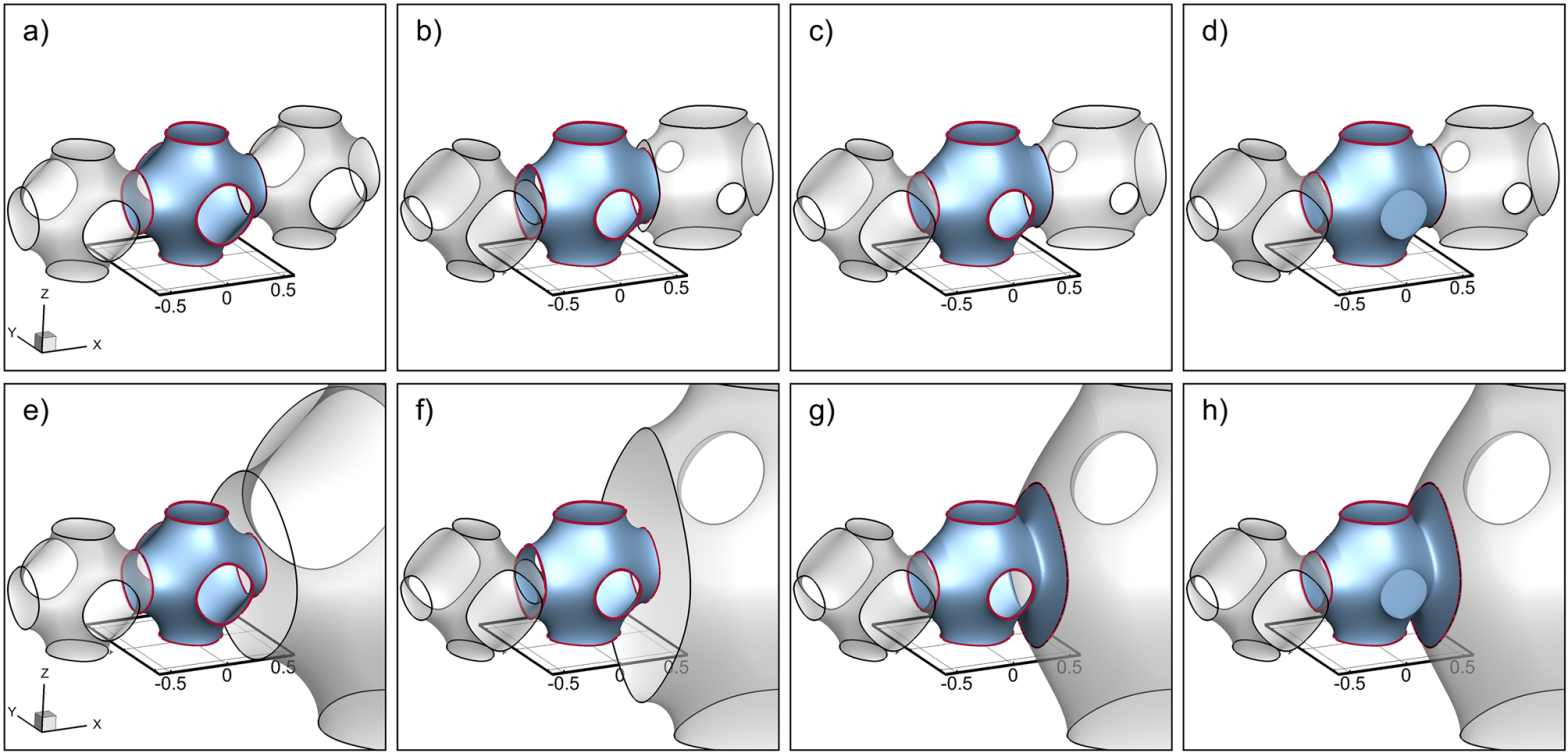
Stepwise creation and manipulation of a single SWP element. Two scenarios are depicted: (**a**–**d**) show SWP element with a same-sized neighboring unit cell and (**e**–**h**) show SWP element with a different sized neighboring unit cell. For both scenarios, the following steps are depicted: (**a**, **e**) initial creation of undeformed SWP element (**b**, **f**) distortion of the SWP element (**c**, **g**) gradual fitting of the connecting interfaces of each SWP element to match neighbor coefficients (if necessary, expand to match larger neighbors) (**d**, **h**) filling interface hole, if no neighbor exists.

After the optimization process had been conducted, a 3D matrix of point-wise permeability values was obtained with the spatial resolution of the computational mesh. These simulated permeabilities were interpolated in Matlab (version 2019a, MathWorks, Natick, MA, USA) so that a permeability value could be obtained for a value at any point within the spatial domain, not simply the points in the computational mesh. Iteratively, a 3 mm × 3 mm × 3 mm region was queried. If the mean value of permeabilities within that region was above the threshold permeability between 1- and 3-mm elements (8.68e−9 m^2^), the quadratic regression equation for 3 mm elements’ permeabilities was used to determine what coefficient for the cosine term in the SWP implicit equation was used to produce that permeability. Alternatively, if the mean permeability fell below the threshold value, a cubic grid of nine 1 mm elements was created, using instead the regression equation for 1 mm elements. An STL mesh for each element was created (Fig. 7a,e) using its calculated cosine coefficient (Fig. 7b,f). As they were, SWP elements with disparate coefficient values or sizes would not fit together at their interface. Therefore, within the first and last eighth of the element’s length in each direction, a transition was defined which adjusted the cosine coefficient and size of the current element to match those of the neighboring element (Fig. 7c,g). If an element was not connected to another element in a certain direction, the SWP-surface was edited to include a face sealing the element in that direction (Fig. 7d,h). The SWP-surface meshes were then exported as STL files. This was repeated for every element that would make up the membrane module.

#### Assembly creation and post-processing

Alongside the individual STL files, the x-, y-, and z-coordinates as well as the size of each element was recorded. This information was then used to import the STL files into a 3-matic (Materialise NV, Leuven, Belgium) project. After translating and scaling each STL file, the single elements were merged into the full membrane module. Due to restrictions regarding the file size of the resulting data, 3-matic’s adaptive remeshing capabilities were used to reduce the mesh complexity before exporting the completed STL file.

#### Manufacturing of the prototype

After preparation of the CAD files had been completed, manufacturing of the individual components and assembly of the prototype device for feasibility and validation purposes could begin. In order to reach the high spatial resolutions required to model the SWP elements, a stereolithography-based 3D printing technique was used to create the membrane modules (Materialise GmbH, Leuven, Belgium). After printing was completed, the remaining resin was washed away from the structure by a combination of pressurized air, a brief soaking in a 50% isopropanol/water solution, and centrifugation. The flow antechambers on either side of the membrane module were manufactured using a material jetting 3D printing technique (Objet 350 Connex3, Stratasys Ltd., Eden Prairie, USA) and VeroClear material.

Other than the removal of the diffuser plates and the consequent thickening of the membrane module to 24 mm to accommodate the empty space, the prototype device remained identical to the predicate.

### Benchtop washout tests

**Figure 8:**
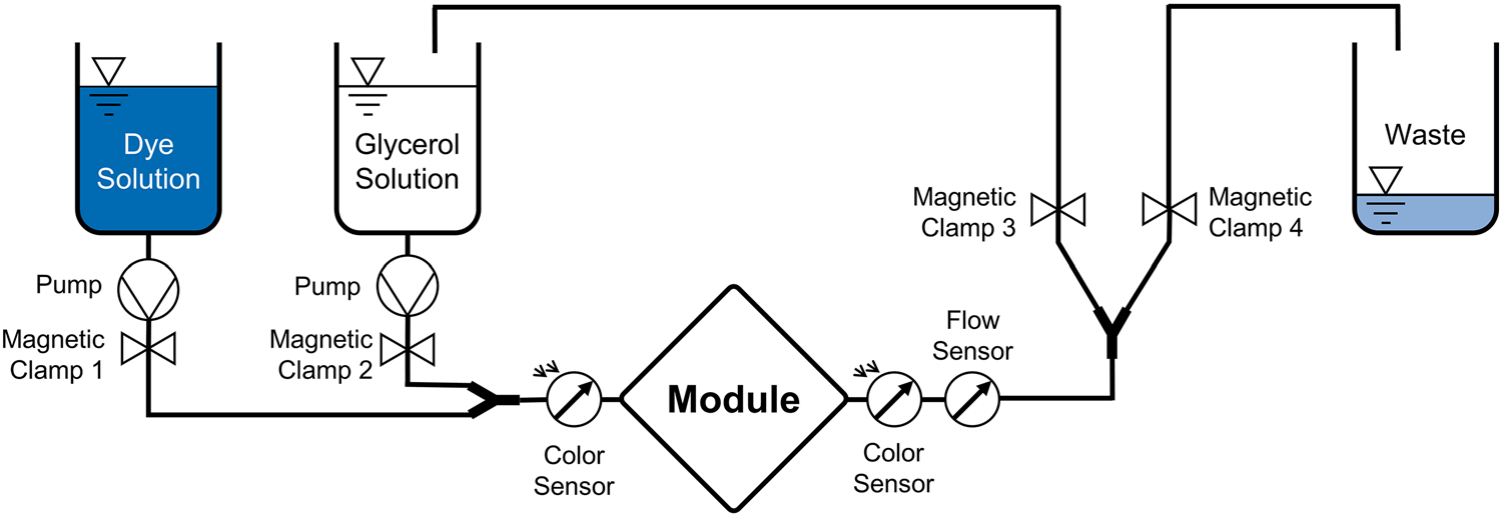
Schematic of the washout test circuit.

In order to validate the simulation results as well as to provide a real-world comparison between the predicate and prototype devices, a series of washout tests was conducted. For the tests, the predicate and prototype devices were measured at the same flow rates as had been simulated. A flow circuit was designed to determine fluid residence times in each device at different flow rates (Fig. 8). The main circuit consists of a glycerol reservoir containing a transparent water glycerol mixture, a pump (deltastream DP 2, Xenios AG, Heilbronn, Germany), and the test module. An additional side arm connected to this circuit in front of the test module including a reservoir with dye solution (water glycerol solution with the same viscous properties as the circulating fluid) and including the same type of pump running at the same operational point. Behind the test module, another side arm leads to a waste reservoir set to the same hydrostatic level as the reservoir with the glycerol solution. A series of magnetic tube clamps (Fluid Concept GmbH, Stutensee, Germany) were positioned on each tubing branch as illustrated in Fig. 8. Initially, the transparent glycerol solution circulated in a closed loop (state of the magnetic tube clamps during ‘circulation phase’: 1 = closed, 2 = open, 3 = open and 4 = closed). The magnetic tube clamps were controlled remotely such that ink could be injected for a prescribed amount of time (state of the magnetic tube clamps during ‘injection phase’: 1 = open, 2 = closed, 3 = closed and 4 = open). During the measurement phase, the polluted fluid was sent to the waste reservoir to keep the remaining fluid transparent (state of the magnetic tube clamps 1 = closed, 2 = open, 3 = closed and 4 = open). After ink was injected, normal flow was resumed. Photometric color sensors (TCS34725, Taos, Inc., Plano, TX, USA) were used to monitor the flow of the injected ink bolus into and out of the module. The photometric sensor readings were continuously recorded for later analysis, and flow rate was monitored with an ultrasonic flow probe (Transonic Systems Inc., Ithaca, NY, USA). Once the dyed fluid without any remains left the circuit, the magnetic tube clamps were reset to the initial circulation phase.

Prior to testing, the photometric color sensors were calibrated with a series of ink-water/glycerol dilutions to obtain a concentration curve. Additionally, flow rate was shown to remain constant during the time when ink was flowing through the module, therefore the mass flow rate of ink could be calculated. From this, threshold values were defined for the minimum, mean, and maximum residence times. Minimum residence time was defined as the time between when 1% of the total dye mass had passed each sensor, mean residence time as the time between the passage of the largest single amount of ink, and maximum residence time as the time between when 95% had passed each sensor.

The predicate and prototype devices were measured at the same flow rates as had been simulated. All tests were conducted using a η = 3.78 ± 0.13 mPas water/glycerol mixture (n = 5 measurements at 0.01–10 Pa, tested via MCR502 rheometer, Anton Paar GmbH, Graz, Austria).

### Ethical approval

This article does not contain any studies with human or animal subjects performed by any of the authors.

## Data Availability

No datasets were generated or analyzed during the current study.

## References

[1] Gabelman A, Hwang S-T (2005). Experimental results versus model predictions for dense gas extraction using a hollow fiber membrane contactor. J. Supercrit. Fluids.

[2] Bazhenov SD, Bildyukevich AV, Volkov AV (2018). Gas-liquid hollow fiber membrane contactors for different applications. Fibers.

[3] Fleming GM (2011). Renal replacement therapy review: Past, present and future. Organogenesis.

[4] Pless G (2007). Artificial and bioartificial liver support. Organogenesis.

[5] Salna M, Bacchetta M (2017). Extracorporeal lung support. Curr. Opin. Anaesthesiol..

[6] Consolo F (2016). On the use of the platelet activity state assay for the in vitro quantification of platelet activation in blood recirculating devices for extracorporeal circulation. Artif. Organs.

[7] Haworth WS (2003). The development of the modern oxygenator. Ann. Thorac. Surg..

[8] Hendrix RHJ, Ganushchak YM, Weerwind PW (2018). Contemporary oxygenator design: Shear stress-related oxygen and carbon dioxide transfer. Artif. Organs.

[9] Tsukiya T (2003). Design progress of the ultracompact integrated heart lung assist device—part 1. Effect of vaned diffusers on gas-transfer performances. Artif. Organs.

[10] Gartner MJ, Wilhelm CR, Gage KL, Fabrizio MC, Wagner WR (2000). Modeling flow effects on thrombotic deposition in a membrane oxygenator. Artif. Organs.

[11] Taga I, Funakubo A, Fukui Y (2005). Design and development of an artificial implantable lung using multiobjective genetic algorithm: Evaluation of gas exchange performance. ASAIO J..

[12] Lehle K (2008). Efficiency in extracorporeal membrane oxygenation-cellular deposits on polymethylpentene membranes increase resistance to blood flow and reduce gas exchange capacity. ASAIO J..

[13] Kaesler A (2018). Technical indicators to evaluate the degree of large clot formation inside the membrane fiber bundle of an oxygenator in an in vitro setup. Artif. Organs..

[14] Lubnow M (2014). Technical complications during veno-venous extracorporeal membrane oxygenation and their relevance predicting a system-exchange–retrospective analysis of 265 cases. PLoS ONE.

[15] Panigada M (2015). Comparison between clinical indicators of transmembrane oxygenator thrombosis and multidetector computed tomographic analysis. J. Crit. Care.

[16] Brogan TV, Thiagarajan RR, Rycus PT, Bartlett RH, Bratton SL (2009). Extracorporeal membrane oxygenation in adults with severe respiratory failure: A multi-center database. Intensive Care Med..

[17] Schlanstein PC (2015). Particle image velocimetry used to qualitatively validate computational fluid dynamic simulations in an oxygenator. A proof of concept. Cardiovasc. Eng. Technol..

[18] Jones CC (2013). Improved computational fluid dynamic simulations of blood flow in membrane oxygenators from X-ray imaging. Ann. Biomed. Eng..

[19] Bachmat Y (1965). Basic Transport Coefficients as Aquifer Characteristics.

[20] Low Z-X (2017). Perspective on 3D printing of separation membranes and comparison to related unconventional fabrication techniques. J. Membr. Sci..

[21] Yazdi AA (2016). 3D printing: An emerging tool for novel microfluidics and lab-on-a-chip applications. Microfluid. Nanofluid..

[22] Gross BC, Erkal JL, Lockwood SY, Chen C, Spence DM (2014). Evaluation of 3D printing and its potential impact on biotechnology and the chemical sciences. Anal. Chem..

[23] Thomas N (2018). 3D printed triply periodic minimal surfaces as spacers for enhanced heat and mass transfer in membrane distillation. Desalination.

[24] Sreedhar N (2018). 3D printed feed spacers based on triply periodic minimal surfaces for flux enhancement and biofouling mitigation in RO and UF. Desalination.

[25] Al-Shimmery A, Mazinani S, Flynn J, Chew J, Mattia D (2019). 3D printed porous contactors for enhanced oil droplet coalescence. J. Membr. Sci..

[26] Femmer T, Kuehne AJC, Wessling M (2015). Estimation of the structure dependent performance of 3-D rapid prototyped membranes. Chem. Eng. J..

[27] Femmer T, Kuehne AJC, Wessling M (2014). Print your own membrane: direct rapid prototyping of polydimethylsiloxane. Lab. Chip..

[28] Femmer T, Kuehne AJC, Torres-Rendon J, Walther A, Wessling M (2015). Print your membrane: Rapid prototyping of complex 3D-PDMS membranes via a sacrificial resist. J. Membr. Sci..

[29] Hesselmann F (2021). Structure-dependent gas transfer performance of 3D-membranes for artificial membrane lungs. J. Membr. Sci..

[30] Melchels FPW (2010). Mathematically defined tissue engineering scaffold architectures prepared by stereolithography. Biomaterials.

[31] Yoo D-J (2011). Computer-aided porous scaffold design for tissue engineering using triply periodic minimal surfaces. Int. J. Precis. Eng. Manuf..

[32] Yoo D-J, Kim K-H (2015). An advanced multi-morphology porous scaffold design method using volumetric distance field and beta growth function. Int. J. Precis. Eng. Manuf..

[33] Zimmermann M (2009). Pumpless extracorporeal interventional lung assist in patients with acute respiratory distress syndrome: A prospective pilot study. Crit. Care.

[34] Toomasian JM (2005). A polymethylpentene fiber gas exchanger for long-term extracorporeal life support. ASAIO J..

[35] Müller T (2009). Extracorporeal pumpless interventional lung assist in clinical practice: Determinants of efficacy. Eur. Respir. J..

[36] Schlanstein PC (2018). Experimental method to determine anisotropic permeability of hollow fiber membrane bundles. J. Membr. Sci..

[37] Kim EJ (2015). Thrombotic complications during interventional lung assist: Case series. Tubercul. Respir. Dis..

[38] Liebold A, Reng CM, Philipp A, Pfeifer M, Birnbaum DE (2000). Pumpless extracorporeal lung assist–experience with the first 20 cases. Eur. J. Cardiothorac. Surg..

[39] Ontaneda A, Annich GM (2018). Novel surfaces in extracorporeal membrane oxygenation circuits. Front. Med..

[40] Guo Y, Liu K, Yu Z (2019). Tetrahedron-based porous scaffold design for 3d printing. Designs.

[41] Yoo D-J (2014). Advanced porous scaffold design using multi-void triply periodic minimal surface models with high surface area to volume ratios. Int. J. Precis. Eng. Manuf..

[42] Arens J (2020). Toward a long-term artificial lung. ASAIO J..

[43] Yoo D-J (2014). Advanced projection image generation algorithm for fabrication of a tissue scaffold using volumetric distance field. Int. J. Precis. Eng. Manuf..

